# Influence of the Addition of Alumina Nanofibers on the Strength of Epoxy Resins

**DOI:** 10.3390/ma16041343

**Published:** 2023-02-04

**Authors:** M. M. Simunin, A. S. Voronin, Yu. V. Fadeev, S. S. Dobrosmyslov, A. A. Kuular, T. A. Shalygina, K. A. Shabanova, D. Yu. Chirkov, S. Yu. Voronina, S. V. Khartov

**Affiliations:** 1 Scientific Laboratory “Smart Materials and Structures”, Reshetnev Siberian State University of Science and Technology, 660037 Krasnoyarsk, Russia; 2 School of Engineering and Construction, Siberian Federal University, 660041 Krasnoyarsk, Russia; 3 Federal Research Center, Krasnoyarsk Science Center SB RAS, 660036 Krasnoyarsk, Russia; 4 Laboratory of EMI Shielding Materials, Bauman Moscow State Technical University, 105005 Moscow, Russia

**Keywords:** alumina nanofibers, epoxy resin, polymer nanocomposites, mechanical strength

## Abstract

The paper describes the effect of the addition of alumina nanofibers on the mechanical properties of the epoxy resin. Alumina nanofibers functionalized with epoxypropyl functional groups are used in this work. The dependence of the mechanical characteristics on the amount of the additive, as well as the features of its distribution in the material, is investigated. In the work, nanocomposites were obtained, which are epoxy resin with aluminum oxide nanofibers. The mechanical properties of the samples were studied by bending tests and differential mechanical analysis (DMA). It has been shown that the addition of alumina nanofibers leads to an increase in ultimate flexural strength. The maximum of this increase is near the percolation threshold of alumina nanofibers in epoxy resin. With the addition of 0.2% alumina nanofibers, the ultimate flexural strength increases from 41 to 71 MPa. It is shown that after exceeding the percolation threshold of nanofibers, the ultimate strength decreases. In this case, the elastic modulus increases from 0.643 to 0.862 GPa. DMA is shown that the glass transition temperature decreases with increasing amount of the additive. This indicates a decrease in the molecular weight of the polymer. By implication, this suggests that the hardener connects the epoxypropyl functional groups on the nanofibers and the epoxy groups in the resin, and as a result of this process, the nanofibers become natural polymer chain length limiters. The data obtained from mechanical testing and differential mechanical analysis can be used to strengthen epoxy resins in polymer composite materials and molding compositions.

## 1. Introduction

Epoxy resins are an important basis for polymer composite materials [1]. Modern industry provides a wide range of such resins from fluid to solid at room temperature. An important property of epoxy resins is their high strength, which, for example, makes it possible to bind carbon fabric or glass fabric into a single composite product that combines high strength and lightness [2]. The strength of polymer composite materials is determined by the fabric base, and the integrity is determined by the resin cohesion energy [3]. A common way to improve the mechanical properties of polymers is to use various mineral fillers, and especially nanomaterials [4]. Usually the shape of the particles used in additives is close to spherical. Such isometric particles can fill up to 74% of the compounded matrix volume [5]. On the other hand, are known many nanomaterials in which the shape of particles is significantly developed to plates (graphene [6], montorillonite [7]), rods (chrysotile [8]) or mobile chains (nanotubes [9] and various linear polymers [10]).

In at least some notable examples, solutions of such highly anisometric particles are known to exhibit significant deviations from Raoult’s law, even going so far as to allow an anisotropic phase to separate from a solution in which the particles occupy no more than 1–2% of the volume of the suspension [11]. This leads to the fact that the addition of anisometric particles to the material can significantly change its properties at very small additions compared to the additions of isometric particles.

An important anisometric additive is alumina nanofibers [12], which increase the cohesive energy of polymeric materials. This leads to an increase in the strength of some polymers [13,14,15] and nanocomposite coatings [16]. However, the addition of nanofibers to polymers by itself cannot be effective without matching the surface of alumina nanofibers with the physicochemical properties of the matrix. A high affinity of the surface of nanofibers and polymer can be achieved using the silanization technology [17], in which a silane with a given functional group is hydrolyzed on hydroxyl groups on the surface of alumina nanofibers.

In general, nanoalumina additives are a well-known strengthening additive. In ceramics, the additive cements and strengthens products [18]. In metals and alloys, the addition of nanoalumina also leads to an increase in the tensile strength of the alloys [19] and an increase in the hardness of the material. Of course, in epoxy resins, nanoalumina fillers also increase the strength and elasticity modulus of the material, both, for adhesive joints [20] and for molded products [21].

In most works, isometric compact additives of alumina nanoparticles are used. However, it is very interesting to study how fiber additives affect the properties of materials. It is clear that in addition to dispersion strengthening, the form of the additive will also provide some reinforcement of the material. The purpose of this work is to strengthen the epoxy resin with the addition of alumina nanofibers. For this, alumina nanofibers functionalized with epoxypropyl functional groups are used in this work. The mechanical properties of samples with different proportions of additives in the resin are studied. As well as the morphology of the distribution of nanofibers in the material.

## 2. Materials and Methods

### 2.1. Materials

Alumina nanofibers were obtained from ENAW-1199 aluminum using the molten aluminum oxidation technology [22], similar to those are used earlier [12,17], obtaining nanofibers having no more than 1% impurities. The silanization process was carried out with silane manufactured by Millipore Sigma, Hamburg, Germany; this is 3-glycidyloxypropyltrimethoxysilane (GlyMS) with a purity of 98%. There also used anhydrous toluene (Sigma-Aldrich Chemie, Taufkirchen, Germany) with a purity of 99.8% as a solvent. GlyMS was used for precipitation of epoxypropyl functional groups. Epoxy resin Araldite 8615 (Huntsman Advanced Materials, Basel, Switzerland) was used as the polymer base of the nanocomposite material with harderer Aradur 8615 (Huntsman Advanced Materials, Basel, Switzerland).

### 2.2. Preparation of Nanocomposite Samples

Alumina nanofibers was coated with epoxypropyl functional groups for better affinity with the epoxy resin. For this, alumina nanofibers were treated in a solution of 3-glycidyloxypropyltrimethoxysilane (98% Evonik, Wesseling, Germany) in toluene (99.8%, Sigma-Aldrich Chemie, Steinheim am Albuch, Germany). For this silanization, 15 g of alumina nanofibers was taken and dried at a temperature of 140 °C in a ShS-40-02 SPU drying oven for about 8 h in order to remove all adsorbed water from the surface, and at the same time to preserve hydroxyl groups in the surface structure. Then, the nanofibers were dispersed in toluene using an overhead stirrer based on the SGR-1 YHCHEM reactor, with a stirring speed of 1000 rpm for 90 min. Next, the suspension was heated to 80 °C with stirring at 600 rpm for 40 min. The silane was added to the obtaining dispersion in the amount of 7 g. After stirring the mixture for an hour, it was cooled, rinsed with toluene, and decanted. Toluene residues were removed by drying at a temperature of 120–140 °C in a drying oven for 8 h.

Alumina nanofibers were added to 180 g of epoxy resin in an amount of 0.12; 0.36; 0.9; 1.8; 3.6 and 7.2 g, then stirred with an OVS-S05 overhead stirrer (Daihan Scientific, Wonju, Korea) for 15 min at 300 rpm, then sonicated (Sonicator 3000, Misonix, Farmingdale, NY, USA) for 5 min. Then the mixture was again alternately stirred mechanically and sonicated. In total, the mixture was treated alternately with a stirrer and ultrasound three times. The resulting suspensions were mixed with 90 g of a hardener, placed into a mold, and evacuated in a desiccator for a 24 h at a residual pressure of 1 mbar. Then the obtained samples were annealed in a ShS-40-02 SPU drying oven at a temperature of 180°C with a heating rate of 1 °C/min. As a result, six samples of nanocomposite material were obtained with a concentration of alumina nanofibers of 0.05; 0.2; 0.5; 1; 2; 4 wt.% and a reference sample without the addition of alumina nanofibers.

### 2.3. Mechanical Tests

The flexural strength was tested according to the method, ISO178:2010. The test bench used was an Instron 3369 testing machine (Instron, High Wycombe, UK). Maximum load 5 tons. The measurement accuracy was about 3%. Linear characteristics were measured using an fit digital caliper with measurement error 0.05%. Nanocomposite materials with different concentrations of alumina nanofibers in the resin were tested. From each nanocomposite material, a series of five plates with dimensions of 3 mm × 10 mm × 35 mm was cut out by circular saw with diamond blade and liquid cooling. Samples were cut out from those parts of the casting where there was no shrinkage gap. Each sample was tested on the test bench and the average value of all tests in each sample was taken as the nanocomposite material test result.

### 2.4. Differential Mechanical Analysis

A thermomechanical study of the viscoelastic properties of the samples was conducted using a dynamic mechanical analyzer Q800 (TA Instruments, New Castle, DE, USA). The studies were carried out according to ISO 6712:1996 (Plastics. Determination of dynamic mechanical properties. Part 5. Flexural vibration. Non-resonance method.) and ISO 6721-11:2012 (Plastics. Determination of dynamic mechanical properties. Part 11. Glass transition temperature). Measurement of storage modulus (E’) of a sample was carried out using a 3-point bending clamp and a linear temperature scanning in the heating mode from 30 to 200 °C at a 5 °C/min rate. The frequency of dynamic loading of the sample was 1 Hz and the relative deformation was less than 0.1%. The samples were investigated in the form of plates with a size of 30 mm × 12.5 mm × 0.65 mm.

### 2.5. Electron Microscopy

The microstructure of the composite films’ cross-sections was studied using scanning electron microscopy (Hitachi SU-3500, Tokyo, Japan) and transmission electron microscopy (Hitachi HT7700, Tokyo, Japan). For scanning electron microscopy (SEM), the pre-treated samples were coated with a thin conductive layer of platinum (at 10 mA, for 45 s) using an EM ACE200 low vacuum coating system (Leica, Teaneck, NJ, USA). For transmission electron microscopy (TEM), the samples were processed Leica EM UC7 ultramicrotome was used to obtain ultrathin sections.

## 3. Results

### 3.1. Morfology of Additive in Resin

**Figure 1:**
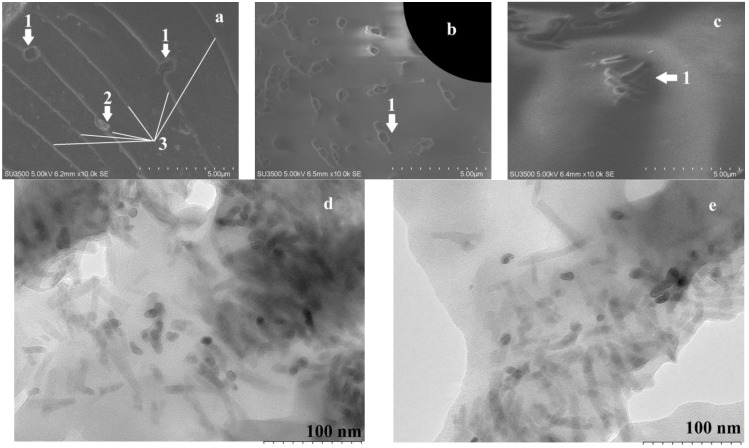
Morphology of the epoxy nanocomposite. (**a**) SEM of chip plane wrinkle texture with inclusions (0.2% alumina), (**b**) SEM of pores on the chip plane (1% alumina), (**c**) SEM of hollows at an angle to the chip plane (0.5% alumina), (**d**) TEM of alumina nanofibers in epoxy resin (4% alumina), and (**e**) TEM of a section of a nanocomposite with pores (2% alumina).

In this work, chip sections of epoxy resin with the addition of alumina nanofibers was studied. SEM shows the characteristic wrinkle texture (Figure 1a, marker 3) associated with mechanical failure of a brittle material. Chips show characteristic inclusions (Figure 1a, marker 2). and holes (Figure 1a–c, markers 1). Inclusions and holes distort the structure of cleavage wrinkles, which indicates the appearance of a difference in the mechanical stress of the free polymer and the marked areas. The holes sometimes take the form of cavities, both perpendicular to the chip sections and at a sharp corner, from which the rods are removed (Figure 1b,c, markers 1). The holes sizes are larger than the average diameter of alumina nanofibers. Nevertheless, these holes should be associated precisely with the removal of alumina nanofibers from the material either in the form of a bundle or in the form of a characteristic conglomerate of nanofibers and a polymer boundary layer around it.

This section may be divided by subheadings. It should provide a concise and precise description of the experimental results, their interpretation, as well as the experimental conclusions that can be drawn.

TEM shows the presence of alumina nanofiber conglomerates (Figure 1d). The additive is spontaneously distributed in the samples, while the interface between the nanofibers and the resin is not clearly distinguished, which indicates the effective wetting of the alumina nanofibers by the resin (Figure 1d,e).

In general, the study of morphology shows that the additive is introduced into the resin, is well affinity by it, and is uniformly distributed over the material. This indicates the effectiveness of attaching epoxypropyl functional groups on the surface of alumina nanofiber and applying such an additive to the epoxy resin. Because, the inclusions and holes distort the structure of cleavage wrinkles indicates that the additive redistributes the mechanical load and prevents material separation during chipping. The fact of nanofibers removal from the polymer during shearing, and not a break at the section site, indicates that the additive exhibits reinforcement properties in the material.

### 3.2. Mechanical Properties of Nanocomposite

According to the results of flexural tests of samples of nanocomposite polymer with different amounts of additives of alumina nanofibers, the following data were obtained (Table 1).

The data indicate an increase in the ultimate flexural stress and elastic modulus of the nanocomposite with an increase in the amount of addition of alumina nanofibers. The local maximum values are achieved with the addition of 0.2 wt.% alumina nanofibers. A further increase in the number of nanofibers leads to a drop in the ultimate stress and elastic modulus of the nanocomposite and a subsequent increase in the elastic modulus while maintaining the ultimate flexural stress. A similar nature of the evolution of the values indicates that, depending on the amount of alumina nanofibers in the material, there are at least two characteristic nanofiber substructures that resist loading. This is obviously associated with the formation of an integral substructure of alumina nanofibers in the polymer by percolation. If for nanofibers with a characteristic aspect ratio equal to 50 to estimate the percolation threshold in epoxy resin, then the known ratios [11,23] will give a value of about 0.25 wt.%. Thus, it can be concluded that the most durable samples are obtained near the percolation threshold. Apparently, a further increase in the amount of the additive leads to coarsening of some regions of the nanofiber substructure. As a result, agglomerates are formed, which, under load, are the centers of crack formation, and, in fact, with an increase in the amount of additive in the polymer, they only become coarser. As a result, the strength of the sample is determined not so much by the distributed substructure of the nanofibers in it, but rather by the strength of the polymer bond between the additive agglomerates, which is observed in samples with high concentrations of the additive. Separately, it should be noted that a further increase in the elastic modulus after a fall when passing through the percolation threshold occurs according to the mixture rule, because a high-modulus aluminum oxide additive is introduced into the polymer. On the other hand, up to the percolation threshold, the increase in the elastic modulus is determined by the elasticity of the distributed substructure of nanofibers. It turns out that up to the percolation threshold, the elasticity of the nanocomposite is affected by the amount of the additive and the structure formed by the nanofibers, and after that, only by the amount of the additive.

### 3.3. Effect of Temperature on the Mechanical Properties of the Nanocomposite

**Figure 2:**
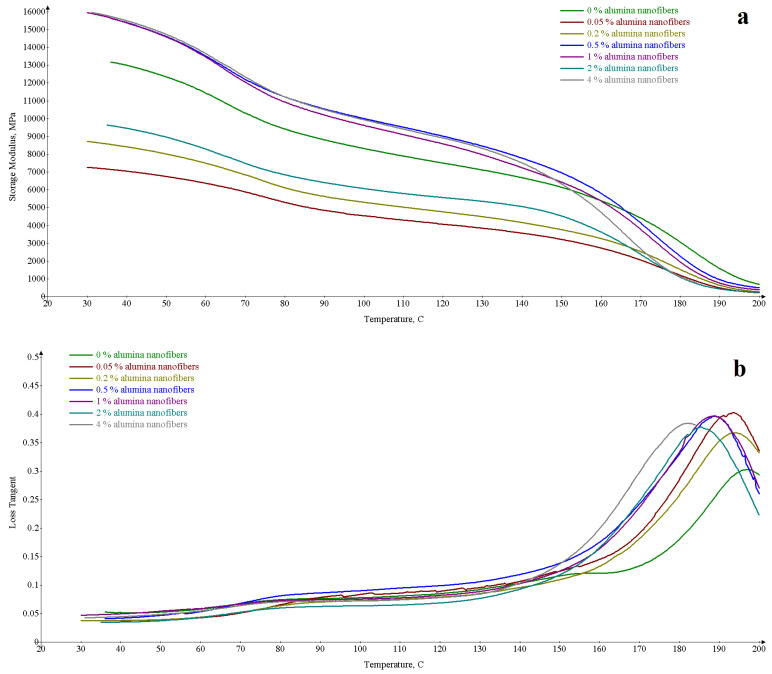

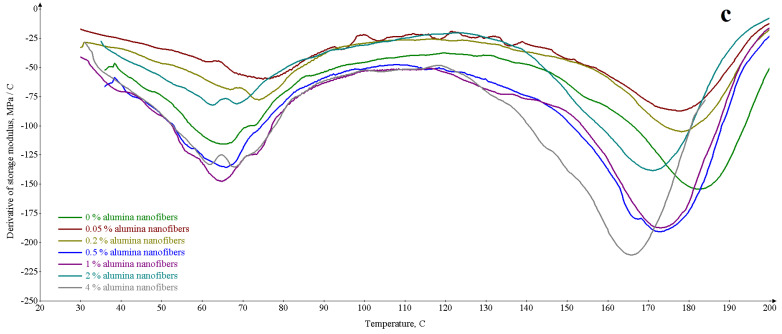
Temperature dependences of dynamic mechanical characteristics of nanocomposites with different amounts of alumina nanofibers. (**a**) Temperature dependences of storage module, (**b**) Temperature dependences of loss tangent, (**c**) Temperature dependences of derivative of storage module.

Differential mechanical analysis allows a more fine-grained study of the substructure of nanofibers in a polymer and provides a rich set of data (Figure 2). The dependence (Figure 2a) of the storage module on temperature is decreasing. It should be noted that the curves of the dependence of the storage modulus on the temperature of the samples with the addition of 0.05% and 0.2% alumina nanofibers lie below the reference, and the graphs of the samples 0.5; 1 and 4% higher, and have approximately the same initial values of the storage module. Separately, the dependence of the storage modulus on the temperature of the sample with the addition of 2% alumina nanofibers is highlighted. The dependence of the mechanical loss tangent on temperature increases. The peaks of the mechanical loss tangent, corresponding to the transition from the glass state to the elastic state, have a temperature (T_max_ loss), the lower, the greater the amount of additive in the polymer. A similar dependence is observed in the graphs for the peaks (T_min_ dE/dT) of the storage module temperature derivative.

The main data from the graphs are summarized in Table 2.

It is clear that the drop in the storage modulus with the addition of alumina nanofibers compared to pure resin is associated with the dispersion of elastic stresses on the inhomogeneities of the nanocomposite material. Indeed, the addition of aluminum oxide nanofibers up to the percolation threshold (0.05%) has the largest loss tangent during the transition to the elastic state, since the scattered nanofibers introduce inhomogeneity into the material. That is, losses occur due to the inhomogeneity of the polymer material and the additive. Losses during the transition to the elastic state of nanocomposites are generally higher than those of pure resin.

The decrease in the glass transition temperature, expressed as a decrease in the temperature of the peaks of the mechanical loss tangent and the derivative of the storage modulus, indicates that with an increase in the number of alumina nanofibers the molecular weight of the polymer, and, consequently, the chain length, decreases. This directly indicates that during the curing of the resin, the polymer chain is terminated due to the presence of nanofibers. By implication, this suggests that the hardener connects the epoxypropyl functional groups on the nanofibers and the epoxy groups in the resin, and as a result of this process, the nanofibers become natural polymer chain length limiters. Here, it should also be noted that the addition of alumina nanofibers does not significantly increase the number of epoxy groups in the resin, since the epoxypropyl groups on the surface of the nanofibers are about 12% [17], and the maximum amount of additive is 4%. It can be roughly estimated that the proportion of epoxy groups in the resin cannot increase by more than 0.5% against a background of 20–22 wt.%. Those additive does not significantly change the ratio of resin and hardener.

The storage modulus above the transition temperature is higher than the viscosity state in a pure sample than with any addition of alumina nanofibers due to dissipative processes due to the inhomogeneity of the material. However, the storage modulus of the sample after the percolation threshold (0.5%) is maximum. This can be explained by the highest uniformity of the integral substructure of the nanofiber. The formation of an integral substructure of nanofibers also has a close value for the storage range for samples with readings of 0.5; 1 and 4% nanofibers. In these samples, the resistance is determined precisely by the substructure of the nanofiber. The reproducible deviation from this generality of samples with 2% additive in the resin is most likely due to the production of highly heterogeneous samples.

The low temperature peaks in the storage modulus derivative plot are associated with residual low molecular weight components in the resin.

## 4. Conclusions

The paper shows the dependence of the mechanical characteristics of epoxy resins on the amount of addition of alumina nanofibers. The maximum ultimate bending strength falls on the amount of additive close to the percolation threshold. The ultimate flexural strength increases from 41 MPa to 71 MPa with the addition of 0.2% alumina nanofibers. In this case, the elastic modulus increases from 0.643 to 0.862 GPa. It is the formation of an integral homogeneous substructure of nanofibers that leads to the maximum strengthening of the polymer. A further increase in the amount of the additive leads to the formation of nanofiber agglomerates and the appearance of inhomogeneities in the nanofiber substructure. The technique of introducing alumina nanofibers into epoxy resin makes it possible to uniformly distribute alumina nanofibers at low additive concentrations.

Based on the presence of epoxypropyl functional groups on the surface of alumina nanofibers, there the hardener crosslinks them with the resin molecules are assumed. This is indirectly indicated by a decrease in the glass transition temperature of the nanocomposite with an increase in the amount of the additive. The morphology of the distribution of the additive and the interface between the nanofibers and the resin shows a high affinity between the polymer matrix and the epoxypropyl-coated alumina nanofibers.

The data obtained can be used to strengthen epoxy resins in polymer composite materials and casting polymer compositions.

As a result, we can formulate the main highlights of the work:

- Obtained nanocomposite samples with different concentrations of alumina nanofibers
- The effectiveness of the use of alumina nanofibers with functionalized epoxypopyl groups has been shown
- To strengthen the epoxy resin from 41 to 71 MPa in bending and from 0.643 to 0.862 GPa, 0.2 wt% of alumina nanofibers is sufficient
- The dependence of the mechanical characteristics of the samples on the proportion of the additive was obtained
- It is shown that with an increase in the number of alumina nanofibers, the glass transition temperature decreases

## Figures and Tables

**Table 1:** Flexural properties of nanocomposites.

Sample	Ultimate Stress	Elastic Modulus
	MPa	GPa
Epoxy reference	41	0.643
Epoxy + 0.05% NFA	53	0.714
Epoxy + 0.2% NFA	71	0.862
Epoxy + 0.5% NFA	56	0.742
Epoxy + 1% NFA	43	0.704
Epoxy + 2% NFA	42	0.801
Epoxy + 4% NFA	42	0.868

**Table 2:** Notable dynamic mechanical characteristics of nanocomposites with different amounts of alumina nanofibers.

Sample	E (T = 40 °C)	E (T = 200 °C)	tg δ_max_	T_max_ loss	T_min_ dE/dT
	MPa	MPa		°C	°C
Epoxy reference	12998	690	0.3029	197	182
Epoxy + 0.05% NFA	7051	222	0.4022	194	178
Epoxy + 0.2% NFA	8418	276	0.3674	194	178
Epoxy + 0.5% NFA	15398	502	0.397	189	173
Epoxy + 1% NFA	15382	391	0.3969	189	173
Epoxy + 2% NFA	9460	250	0.3773	185	171
Epoxy + 4% NFA	15500	243	0.3843	182	166

## Data Availability

Not applicable.
